# Overexpression of amplified in breast cancer 1 (*AIB1*) gene promotes lung adenocarcinoma aggressiveness in vitro and in vivo by upregulating C-X-C motif chemokine receptor 4

**DOI:** 10.1186/s40880-018-0320-1

**Published:** 2018-08-13

**Authors:** Liru He, Haixia Deng, Shiliang Liu, Jiewei Chen, Binkui Li, Chenyuan Wang, Xin Wang, Yiguo Jiang, Ningfang Ma, Mengzhong Liu, Dan Xie

**Affiliations:** 1 The State Key Laboratory of Oncology in South China, Collaborative Innovation Center for Cancer Medicine, Sun Yat-Sen University Cancer Center, No. 651, Dongfeng Road East, Guangzhou, 510060 China; 2 Department of Radiation Oncology, Sun Yat-Sen University Cancer Center, Guangzhou, China; 3 Department of Thoracic Oncology, Sun Yat-Sen University Cancer Center, Guangzhou, China; 4 The State Key Laboratory of Respiratory Disease, Guangzhou Medical University, Guangzhou, China; 5 Key Laboratory of Protein Modification and Degradation, School of Basic Medical Sciences, Affiliated Cancer Hospital & Institute of Guangzhou Medical University, Guangzhou, China

**Keywords:** Lung adenocarcinoma, Amplified in breast cancer 1, C-X-C motif chemokine receptor 4, Metastasis, Prognosis

## Abstract

**Background:**

We previously found that overexpression of the gene known as amplified in breast cancer 1 (*AIB1*) was associated with lymph node metastasis and poor prognosis in patients with lung adenocarcinoma. However, the role of AIB1 in that malignancy remains unknown. The present study aimed to investigate the function of AIB1 in the process of lung adenocarcinoma cell metastasis.

**Methods:**

A series of in vivo and in vitro assays were performed to elucidate the function of AIB1, while real-time PCR and Western blotting were utilized to identify the potential downstream targets of AIB1 in the process of lung adenocarcinoma metastasis. Rescue experiments and in vitro assays were performed to investigate whether the invasiveness of AIB1-induced lung adenocarcinoma was mediated by C-X-C motif chemokine receptor 4 (CXCR4).

**Results:**

The ectopic overexpression of AIB1 in lung adenocarcinoma cells substantially enhanced cell migration and invasive abilities in vitro and tumor metastasis in vivo, whereas the depletion of AIB1 expression substantially inhibited lung adenocarcinoma cell migration and invasion. CXCR4 was identified as a potential downstream target of AIB1 in lung adenocarcinoma. The knockdown of AIB1 greatly reduced CXCR4 gene expression at both the transcription and protein levels, whereas the knockdown of CXCR4 in cells with AIB1 ectopic overexpression diminished AIB1-induced migration and invasion in vitro and tumor metastasis in vivo. Furthermore, we found a significant positive association between the expression of AIB1 and CXCR4 in lung adenocarcinoma patients (183 cases), and the co-overexpression of AIB1 and CXCR4 predicted the poorest prognosis.

**Conclusions:**

These findings suggest that AIB1 promotes the aggressiveness of lung adenocarcinoma in vitro and in vivo by upregulating CXCR4 and that it might be usable as a novel prognostic marker and/or therapeutic target for this disease.

**Electronic supplementary material:**

The online version of this article (10.1186/s40880-018-0320-1) contains supplementary material, which is available to authorized users.

## Background

Lung cancer is responsible for the most cancer-related morbidity and mortality worldwide [1, 2], and lung adenocarcinoma is the major histologic subtype of lung cancer. Despite considerable therapeutic progress, the prognosis of patients with lung adenocarcinoma (LA) remains very poor [3], and metastasis is the main cause of cancer death [4]. It is known that the metastatic process of lung adenocarcinoma is due to multiple molecular abnormalities, such as the activation of numerous important oncogenes and/or inactivation of various tumor suppressor genes [5]. Therefore, a better understanding of the biological mechanisms underlying the metastasis of lung adenocarcinoma is crucial for the discovery of novel therapeutic targets and the consequent improvement of cancer treatment [6].

The amplified in breast cancer 1 (*AIB1*) gene was initially reported to be involved in a number of biological processes, including cell differentiation, proliferation, survival and migration in hormone-sensitive cancers [7, 8]. We recently reported that AIB1 was also overexpressed and closely correlated with advanced clinical stages and/or poor prognoses in a series of hormone-insensitive malignancies [9–14], including lung adenocarcinoma [13]. Our data suggest a potential selective advantage of AIB1 in promoting the lymph node metastasis of lung adenocarcinoma [13]. Very recently, Mo et al. [15] reported that AIB1 promotes colorectal cancer metastasis by enhancing Notch signaling. These data suggest that AIB1 may also be an important oncogene involved in tumor metastasis in hormone-insensitive cancers.

To date, only some signaling pathways, such as the matrix metalloproteinase (MMP) [16, 17], focal adhesion kinase (FAK) [18] and Notch signaling pathways [15], have been identified as molecular mechanisms by which AIB1 promotes cancer cell metastasis. C-X-C motif chemokine receptor 4 (CXCR4), which plays an important role in the cell proliferation and metastasis of lung adenocarcinoma, has also been demonstrated to be a transcriptional target of AIB1 involved in promoting cell proliferation in breast and bladder cancers [19, 20]. However, it is unknown whether CXCR4 is a functional downstream target in aggressive AIB1-mediated lung adenocarcinoma.

To elucidate the potential role of *AIB1* in the development of lung adenocarcinoma, we investigated the function and underlying molecular mechanisms by which *AIB1* mediates tumor cell metastasis in lung adenocarcinoma cell lines.

## Methods

### Patients and tissue specimens

Thirty pairs of lung adenocarcinoma and their adjacent normal tissue samples (10 localized, 10 regional and 10 metastatic cases) were obtained with informed consent under institutional review board-approved protocols between January 2012 and December 2012 from Sun Yat-sen University Cancer Center, Guangzhou, China. Tumors without regional lymph nodes or distant metastases, tumors with regional lymph node metastases but without distant metastases, and tumor with distant metastases were defined as localized, regional and metastatic cases, respectively. Paraffin-embedded pathological specimens from 183 lung adenocarcinoma patients treated between October 1994 and February 1998 were obtained from the archives of the Department of Pathology at the same institution. All the patients were treated with initial surgical resection with a curative or palliative intent. The cases were selected consecutively based on the availability of resection tissue and follow-up data. Tumor differentiation grades and pathological tumor-node-metastasis (TNM) status were assessed according to the criteria of the World Health Organization and the 8th edition of the TNM classification of the International Union Against Cancer (UICC, 2015). The medical ethics committee of the Cancer Center of Sun Yat-sen University approved this study.

### Construction of tissue microarrays (TMAs)

TMAs were constructed according to the method described previously [21]. The tissues (183 lung adenocarcinomas and 30 normal lung tissues from the same patients) were sampled using a tissue arraying instrument (Beecher Instruments, Silver Spring, MD, USA).

### Immunohistochemistry (IHC)

Endogenous peroxidase activity was blocked with 0.3% hydrogen peroxide for 15 min. Tissue slides were boiled in 10 mmol/L citrate buffer (pH 6.0) (Beyotime, Shanghai, China) in a pressure cooker for 10 min (AIB1) or microwave-treated for 10 min for antigen retrieval. The slides were incubated with anti-AIB1 [Clone 34, BD Transduction Laboratories, San Jose, CA, USA, diluted 1:50 in phosphate buffer saline (PBS)] and anti-CXCR4 (Clone 2074, Abcam, Cambridge, UK, diluted 1:1000 in PBS) overnight at 4 °C. Subsequently, the slides were sequentially incubated with biotinylated rabbit antimouse immunoglobulin (Dako, Carpinteria, CA, USA) at a concentration of 1:100 for 30 min at 37 °C and then reacted with a streptavidin-peroxidase (Dako) conjugate for 30 min at 37 °C and 3′-3′ diaminobenzidine (Dako) as a chromogen substrate. The nucleus was counterstained using Meyer’s hematoxylin (Sigma, St. Louis, MO, USA).

Since the positive nuclei staining of normal lung tissues ranged from 0% to 10% of the epithelium, normal expression and overexpression of AIB1 were identified when the nuclei of ≤ 10% and > 10% of tumor cells were positively stained, respectively. To evaluate CXCR4 IHC staining, a previously validated semi-quantitative scoring criterion was used [22, 23]. A staining index (values 0–9) was calculated by multiplying a score reflecting the intensity of CXCR4-positive staining (negative = 0, weak = 1, moderate = 2, and strong = 3) and a score reflecting the proportion of immunopositive cells of interest (< 10% = 1, 10% to 50% = 2, and > 50% = 3.

### Cell lines and culture conditions

Four lung adenocarcinoma cell lines (A549, H1975, H2073 and PC9) were cultured in RPMI1640 (Gibco, Grand Island, NY, USA) medium with 10% newborn calf serum. (Gibco, Grand Island, NY, USA) Another lung adenocarcinoma cell line, H1993, was maintained in Dulbecco’s modified Eagle’s medium supplemented with 10% fetal bovine serum (FBS) (Gibco). All 5 cell lines were obtained from the American Type Culture Collection (ATCC, Manassas, VA, USA).

### Protein extraction and Western blotting

The protein was extracted from the lung adenocarcinoma cells using Radio-Immunoprecipitation Assay (RIPA) Lysis Buffer (Beyotime) at 4 °C. Protein concentrations were measured by the Bicinchoninic Acid Protein Assay (BioRad, Hercules, CA, USA). Equal amounts of whole-cell lysates were resolved by sodium dodecyl sulfate-polyacrylamide gel electrophoresis and transferred onto a polyvinylidene difluoride membrane (Millipore, Bedford, MA, USA) followed by incubation with primary mouse monoclonal antibodies against human AIB1 (1:1000 dilution), CXCR4 (1:500 dilution), tumor necrosis factor (ligand) superfamily member 10 (TNFSF10) (1:500 dilution), matrix metallopeptidase 11 (MMP11) (1:1000 dilution), matrix metallopeptidase 2 (MMP2) (1:500 dilution), and vascular endothelial growth factor A (VEGFA) (1:1000 dilution) (BD Transduction Laboratories) overnight at 4 °C. β-Actin was used as an internal control (1:1000 dilution, BD Transduction Laboratories). After washing, the polyvinylidene fluoride (PVDF) membranes were incubated with secondary antibody (goat anti-mouse, 1:10,000 dilution, Cell Signaling Technology, Danvers, MA, USA) for 2 hat room temperature. The immunoreactive proteins were detected with enhanced chemiluminescence detection reagents (Amersham Biosciences, Uppsala, Sweden) according to the manufacturer’s instructions.

### Knockdown of AIB1 and CXCR4 by lentiviral short hairpin RNA (shRNA)

We synthesized the sequences of AIB1 to construct lentiviral shRNA1 (5′-GGTCTTACCTGCAGTGGTGAA-3′) and shRNA2 (5′-AGACTCCTTAGGACC GCTT-3′), which have been previously found to efficiently knock down endogenous AIB1 expression in human cancer cells [14]. The shRNA sequence for CXCR4 is 5′-ACCGCGATCAGTGTGAGTATATAAAGTTCTCTTATATACTCACACTGATCGCTTTTTC-3′, which was also previously validated [24]. Virus packaging was performed by the transient transfection of 293FT cells with a transfer plasmid and three packaging plasmids: pMDLg/pRRE, pRSV-REV, and pCMV-VSVG, which were kindly provided by Professor Peng Xiang (Center for Stem Cell Biology and Tissue Engineering, Sun Yat-sen University). Seventy-two hours after transfection, the lentiviral particles were collected, filtered, and then concentrated. Subsequently, we infected the lung adenocarcinoma cell lines with the lentivirus in a 24-well plate. Four days after infection, the knockdown efficiency was examined by Western blotting.

### Plasmid constructs and transfection

The construction of a plasmid expressing human AIB1 (pcDNA-AIB1) was conducted as described in our previous study [25]. In brief, full-length human AIB1 cDNA was amplified by PCR (primer: 5′-GTCATATGATGAGTGGATTAGGAGAAAAC -3′ (forward) and 5′- CGAGATCTTCAGCAGTATTTCTGATCAGG-3′ (reverse), initial denaturation at 95 °C for 10 min and 35 cycles of 95 °C for 15 s, 55 °C for 30 s, and 72 °C for 5 min) and cloned into the NheI and EcoRI site pcDNA3.1 (+) expression vector (Invitrogen, Carlsbad, CA), then transfected into A549 cells using Lipofectamine 2000 (Invitrogen, Carlsbad, CA) according to the manufacturer’s instructions. Cells transfected with empty vector were used as controls. Stable AIB1-expressing clones were selected by Geneticin (Rache Diagnostics, Indianapolis, IN) (500 μg/mL).

### RNA interference (RNAi)

Short interfering RNAs (siRNAs) specifically directed against the CXCR4 gene (1: 5′-AUCACGUAAAGCUAGAAA-3′, 5′-GGGAUCAUUUCUAGCUUU-3′; 2: 5′-GCUGUUUAUGCAUAGAUA-3′, 5′-GAGAGAUUAUCUAUGCAU-3′) [26] and corresponding scrambled siRNAs (Ribo bio, Guangzhou, China) were transfected into A549 cells in six-well plates using Lipofectamine 2000 transfection reagent (Invitrogen) according to the manufacturer’s instructions.

### Migration and invasion assays

Cell migration was assessed by measuring the movement of cells into a scratch created by a 200 ml pipette tube. The degree of wound closure was observed after 24 h and photographed under a microscope. The fraction of cell coverage across the line was measured to determine the migration rate. Wound repair = [(Diameter of the wound before migration − Diameter of the wound after migration)/Diameter of the wound before migration] × 100%. Each independent experiment was repeated three times.

For invasion assays, cells (3 × 10^5^) were added to a Matrigel invasion chamber (BD Biosciences, Becton Dickson Labware, Flanklin Lakes, New Jersey, USA) in the insert of a 24-well culture plate. Fetal bovine serum was added to the lower chamber as a chemoattractant. After 24 h, invasive cells located on the lower side of the chamber were fixed and stained with crystal violet, air dried, and photographed. The invasive cells were counted in five fields under an inverted microscope. Experiments were performed in triplicate with a minimum of 40 grids (400 magnification) per filter counted.

### Real-time PCR

RNA was extracted from H1993 AIB1_shRNA2 and H1993_control_shRNA using Trizol (Invitrogen) and was cleaned using the RNeasy MinElute Cleanup Kit (Qiagen, Valencia, California, USA). The concentrations of the RNA samples were measured by NanoDrop 2000 (Thermo Fisher Scientific, Waltham Massachusetts, US). Subsequently, total RNA was reverse transcribed using Super- Script III reverse transcriptase (Invitrogen), and cDNA was amplified by PCR using 2×Super Array PCR Master Mix (SuperArray Bioscience, Frederick, Maryland, USA). Real-time PCR was then performed on each sample using the Human Tumor Metastasis RT2 ProfilerPCR array (SuperArray Bioscience) in an Opticon DNA Engine ABI PRISM7900 system (Applied Biosystems, Foster City, CA, USA), according to the manufacturer’s instructions. Data were normalized to glyceraldehyde phosphate dehydrogenase (GAPDH) levels by the ∆∆Ct method [27].

### In vivo metastasis model

Animal experiments were carried out in accordance with the National Institutes of Health Guide for the Care and Use of Laboratory animals (NIH Publications No. 8023, revised 1978). Eight 4-week-old Balb/c nude mice, which were purchased from Shanghai Slac Laboratory Animal Co. Ltd. (Shanghai, China), were injected with A549-Vec, A549-AIB1, or A549-AIB1 + CXCR4 shRNA cells. Briefly, 2 × 10^5^ cells (mixed with 100 μL PBS) were injected intravenously through the tail vein into each Balb/c nude mouse in a laminar flow cabinet. Six weeks after cell injection, the mice were killed by cervical dislocation. Their livers and lungs were harvested, fixed in 4% paraformaldehyde, and embedded in paraffin. Subsequently, serial 2-μm-thick sections of the whole lungs and livers were obtained and examined by hematoxylin and eosin (H&E) staining to identify the metastases. All the procedures were performed in accordance with the guidelines of the laboratory animal ethics committee of Sun Yat-sen University.

### Statistical analysis

Statistical analysis was performed with SPSS software (SPSS Standard version 19.0, SPSS Inc. Chicago, IL). Receiver operating characteristic (ROC) curve analysis was applied to determine the cutoff scores of AIB1 expression for distinguishing localized, regional, and metastatic lung adenocarcinomas. The sensitivity, specificity, and areas under the ROC curves (AUC) were calculated. The association of AIB1 protein expression with clinicopathologic features and the correlations between molecular features were assessed by the Chi square test. Survival curves were assessed by the Kaplan–Meier method and compared by the log-rank test. Two-sided *P* values of less than 0.05 were considered to indicate statistical significance.

## Results

### IHC staining of AIB1 expression in the localized, regional, and metastatic stages of lung adenocarcinoma tissues

**Fig. 1:**
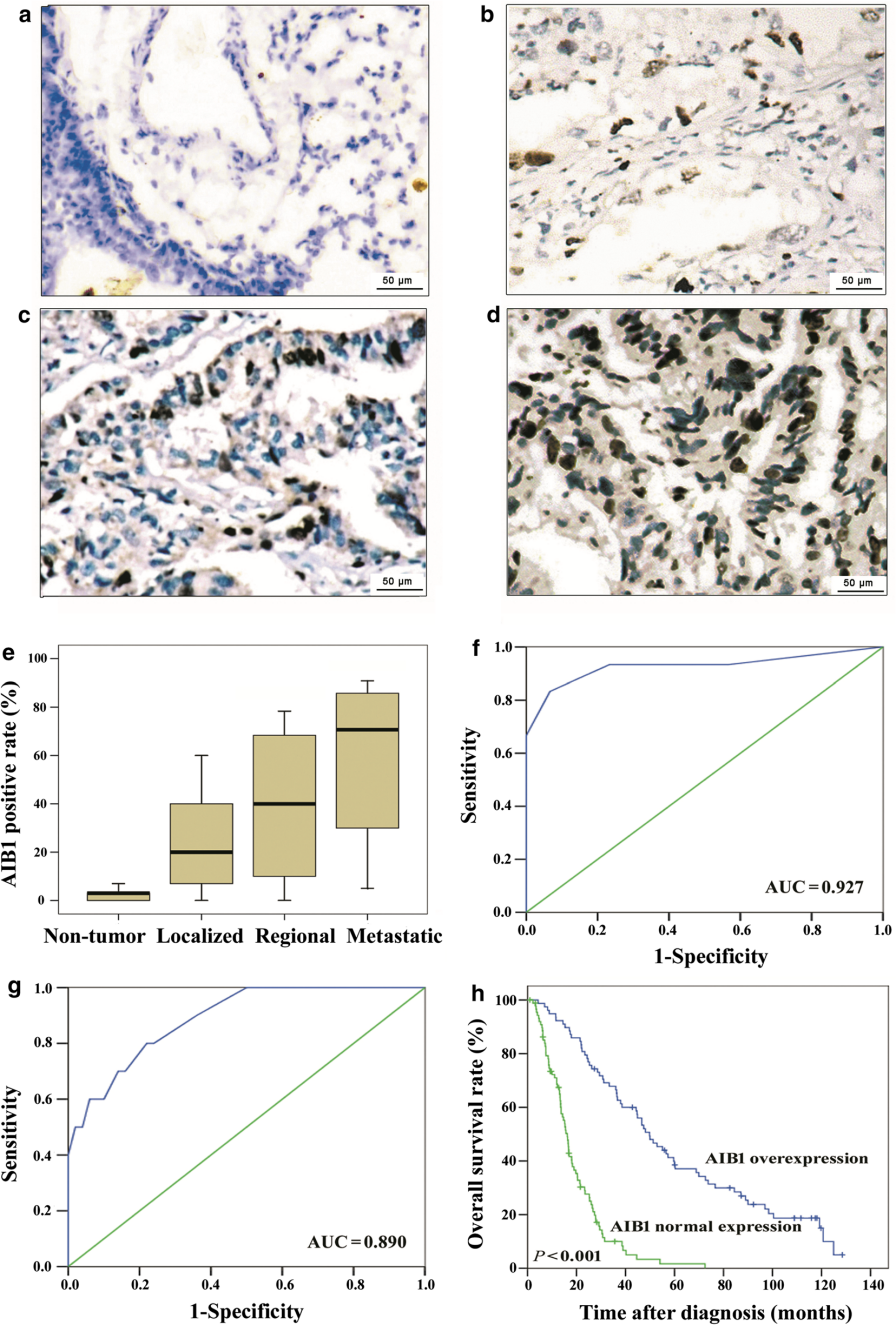
Amplified in breast cancer 1 (AIB1) expression in lung adenocarcinoma tissues. Immunohistochemical (IHC) staining of AIB1 in normal lung tissues (**a**) and the primary lesions of localized (n = 61, mean positive rate of AIB1 expression is 19.7%) (**b**), regional (n = 76, mean positive rate of AIB1 expression is 40.3%) (**c**), and metastatic (n = 30, mean positive rate of AIB1 expression is 70.5%) (**d**) lung adenocarcinoma tissues. **e** The box plots demonstrate the range of AIB1 expression within each group (non-tumor, localized tumor, regional tumor, and metastatic tumor). **f** Receiver operating characteristic (ROC) curve of AIB1 in lung adenocarcinomas with regional lymph node metastasis compared to non-regional metastatic lung adenocarcinomas. Blue line: ROC curve, green line: reference line. **g** ROC curve of AIB1 in metastatic lung adenocarcinomas compared to non-metastatic lung adenocarcinomas. Blue line: ROC curve, green line: reference line. **h** Overall survival curve according to AIB1 expression level for 167 lung adenocarcinoma patients

The mean positive rates of AIB1 expression in lung adenocarcinoma at the localized, regional, and metastatic stages of lung adenocarcinoma were about 20.0%, 40.0%, and 70.0% respectively, compared with 3.0% (range 0.0%–10.0%) for normal tissues (*P *< 0.001, Fig. 1a–e). The sensitivity, specificity, and area under the ROC curve (AUC) values of AIB1 expression levels for regional versus nonregional stages were 83.3%, 93.3%, and 0.927, respectively (*P *< 0.001, Fig. 1f), whereas the sensitivity, specificity, and AUC values of AIB1 expression levels for metastatic versus nonmetastatic stages were 80.0%, 78.0%, and 0.890, respectively (*P *< 0.001, Fig. 1g).

### Association between the expression of AIB1 and lung adenocarcinoma patient clinicopathologic features and survival

We evaluated AIB1 expression in 167/183 (91.3%) of lung adenocarcinomas and 27/30 (90.0%) of normal lung tissues; lost samples, samples with too few tumor cells (< 300 cells per case), and unrepresentative samples were not used in data compilation. Employing the previously described criterion (normal expression and overexpression of AIB1 were identified when at least 10% and more than 10%, respectively, of tumor cell nuclei were positively stained), overexpression of AIB1 was observed in 41.3%, 60.6%, 47.4%, and 80% of samples in the N0, N+, M0, and M1 stages of lung adenocarcinoma, respectively (Table 1). Overexpression of AIB1 was correlated with an ascending clinical stage (*P *< 0.001, Table 1) and poor survival in lung adenocarcinoma patients (*P *< 0.001, Fig. 1h).

**Table 1:** Amplified in breast cancer 1 (AIB1) expression status and characteristics of the 167 lung adenocarcinoma patients

Characteristic	Total (cases)	AIB1 expression [cases (%)]		*P*
		Normal expression	Overexpression	
Age (years)				0.959
≤ 50^a^	81	38 (46.9)	43 (53.1)	
> 50	86	40 (46.5)	46 (53.5)	
Gender				0.704
Male	118	54 (45.8)	64 (54.2)	
Female	49	24 (49.0)	25 (51.0)	
Tumor grade				0.110
G1	41	23 (56.1)	18 (43.9)	
G2	82	40 (48.8)	42 (51.2)	
G3	44	15 (34.1)	29 (65.9)	
T status				0.226
T1–2	88	45 (51.1)	43 (48.9)	
T3–4	79	33 (41.8)	46 (58.2)	
N status				0.015
N0	63	37 (58.7)	26 (41.3)	
N1–3	104	41 (39.4)	63 (60.6)	
M status				0.001
M0	137	72 (52.6)	65 (47.4)	
M1	30	6 (20.0)	24 (80.0)	
Stage				< 0.001
I	32	24 (75.0)	8 (25.0)	
II	28	17 (60.7)	11 (39.3)	
III	77	31 (40.3)	46 (59.7)	
IV	30	6 (20.0)	24 (80.0)	

^a^Median age = 50 years old

### Silencing of AIB1 by RNA interference inhibits lung adenocarcinoma cell migration and invasion in vitro

**Fig. 2:**
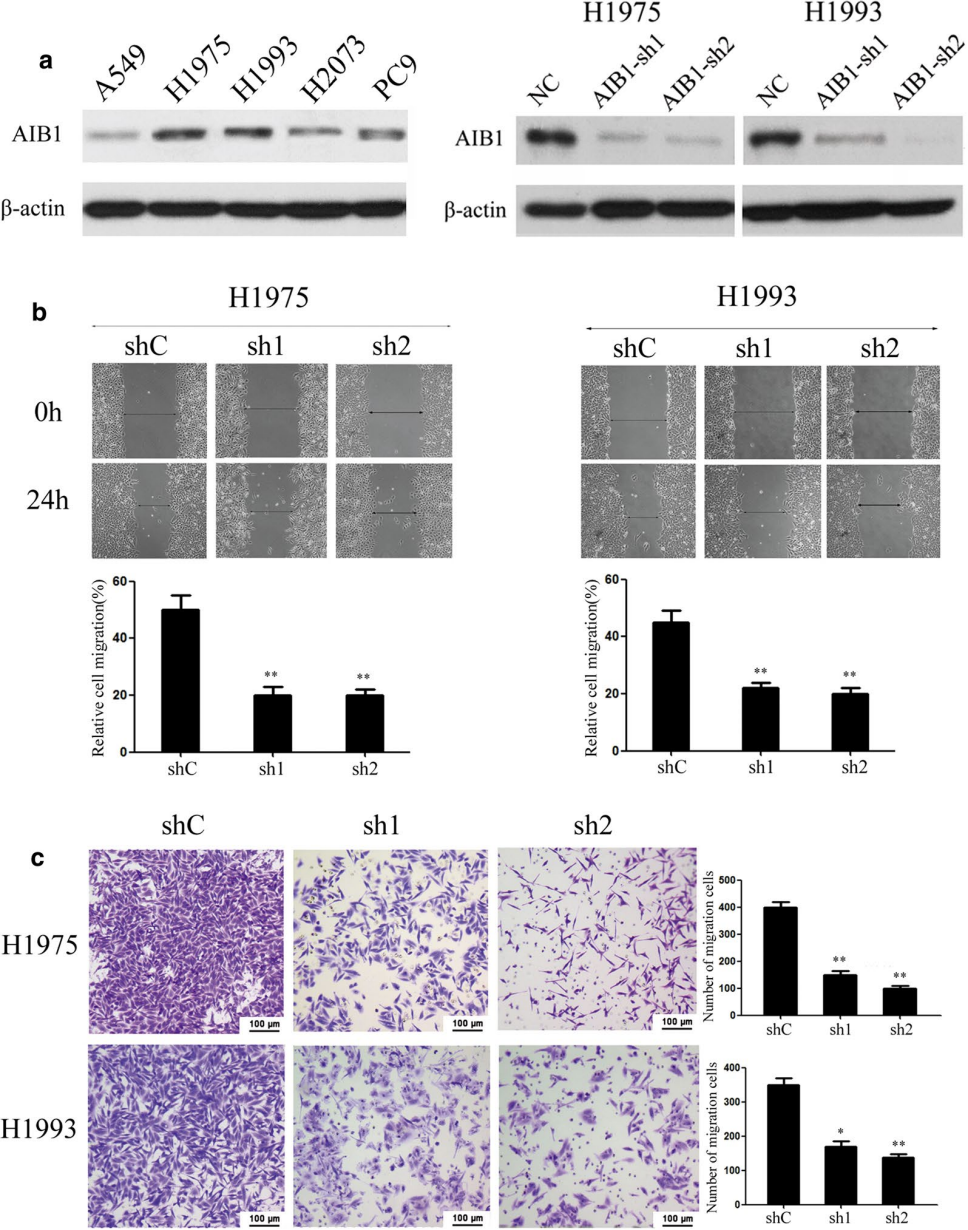
Silencing of AIB1 by RNA interference inhibits lung adenocarcinoma cell migration and invasion in vitro. **a** Left: the levels of AIB1 expression in 5 lung adenocarcinoma cell lines by Western blotting analysis; right: Western blotting reveals that AIB1 was efficiently knocked down by the treatment with AIB1-shRNA1 and AIB1-shRNA2. **b** Wound-healing assays show that AIB1-silenced H1975 and H1993 cells had lower motility than control cells. **c** Cell invasion was evaluated using a Matrigel invasion chamber. Silencing of AIB1 decreased H1975 and H1993 cell invasive capacity. The numbers of invaded cells in the shAIB1 and control groups are shown in the right panel. Data are the mean ± standard error (SE) of three independent experiments; *P < 0.05, **P < 0.01 versus cells transfected with shC by Student’s t test

Of the five lung adenocarcinoma cell lines analyzed, H1975, H1993, H2073, and PC9 cells showed relatively high levels of endogenous AIB1 protein expression, whereas A549 cells showed relatively low levels of AIB1 protein expression (Fig. 2a left).Two lung adenocarcinoma cell lines, H1975 and H1993, were then treated with two specific shRNAs against AIB1, and the shRNAs could efficiently knock down endogenous AIB1 in lung adenocarcinoma cells (Fig. 2a right). The knockdown of AIB1 caused an apparent suppression of cell migration in both H1975 and H1993 cell lines, as shown by using a wound-healing assay (*P *< 0.01, Fig. 2b). The ablation of endogenous AIB1 markedly reduced the invasive ability of both H1975 and H1993 cell lines in Matrigel invasion assays (*P *< 0.05, Fig. 2c).

### Ectopic overexpression of AIB1 by plasmid transfection promotes lung adenocarcinoma cell migration and invasion in vitro

**Fig. 3:**
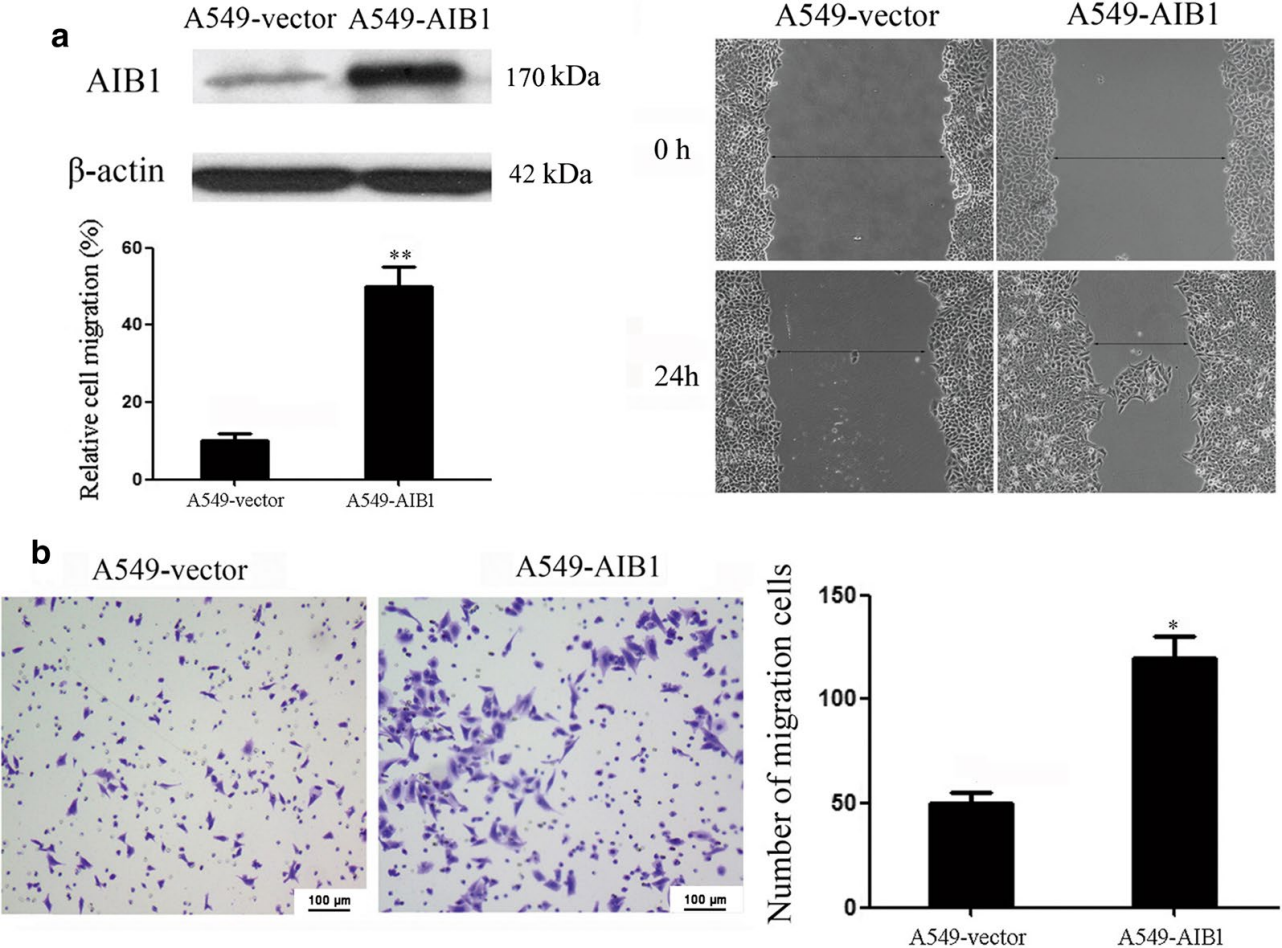
Overexpression of AIB1 enhances lung adenocarcinoma A549 cell migration and invasion in vitro. **a** Left: Western blotting reveals that ectopic expression of AIB1 was substantially increased in A549-AIB1 cells compared with that in A549-vector cells (upper panel); right: representative results of wound-healing assays demonstrate that A549-AIB1 cells had higher motility than A549-vector cells. The numbers of migrating cells are shown in the left bottom panel. Data are the mean ± SE of three independent experiments; **P < 0.001 by Student’s t test. **b** Ectopic overexpression of AIB1 enhanced A549 cell invasion in a Transwell assay. Data are the mean ± SE of three independent experiments; *P < 0.05 by Student’s t test

To determine whether the ectopic overexpression of AIB1 could enhance the migration and invasion capacity of lung adenocarcinoma cells, the A549-AIB1 cell line, which overexpressed AIB1, was constructed and used to perform wound-healing and invasion assays (Fig. 3a left) The wound-healing assay showed that the ectopic overexpression of AIB1 enhanced A549 cell migration at the edge of the exposed regions (*P *< 0.01, Fig. 3a right). The Matrigel invasion assay demonstrated that the invasive capacity of the A549-AIB1 cells was greater than that of the control A549-vector cells (*P *< 0.05, Fig. 3b).

### AIB1 up-regulates CXCR4 expression in lung adenocarcinoma cells

**Fig. 4:**
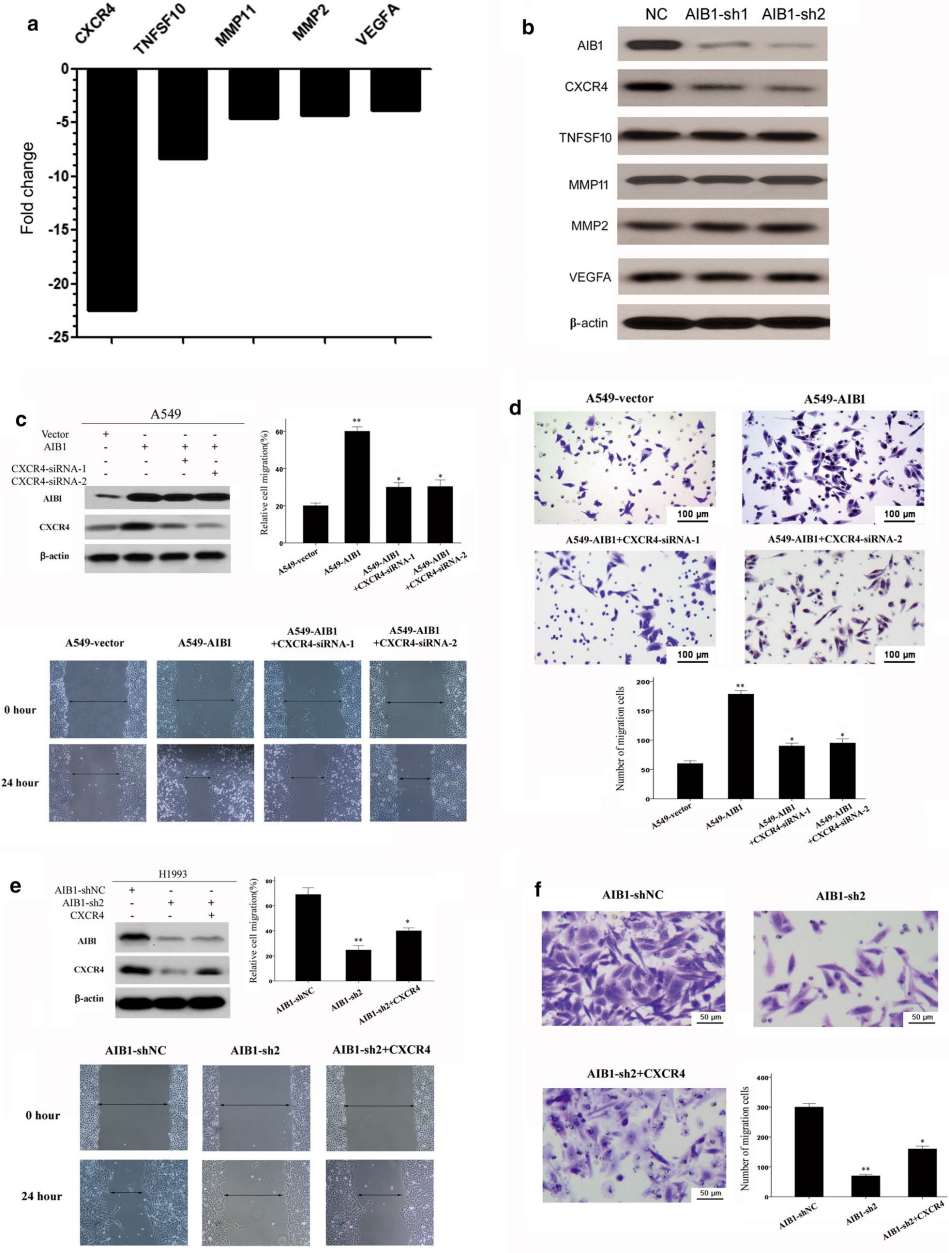
The associations of AIB1 and C-X-C motif chemokine receptor 4 (CXCR4) expression in lung adenocarcinoma cells. **a** The five genes, CXCR4, tumor necrosis factor (ligand) superfamily member 10 (TNFSF10), matrix metallopeptidase 11 (MMP11), matrix metallopeptidase 2 (MMP2), and vascular endothelial growth factor A (VEGFA), showed more than a 3.5-fold mRNA differential expression in shAIB1-transfected H1993 cells compared with that in control H1993 cells, as shown by using a human tumor metastasis RT2 profiler PCR array. **b** Silencing of AIB1 by two shRNAs down-regulated CXCR4 expression in shAIB1 H1993 cells, as detected by Western blotting. **c** Upper left: treatment of 2 CXCR4-shRNAs in A549-AIB1 cells efficiently decreased the expression levels of CXCR4 as detected by Western blotting. Upper right and down: wound-healing assay showed that the enhanced migrative ability in A549-AIB1 cells was inhibited by silencing CXCR4. **d** Transwell assay demonstrated that the increased invasive capacity of A549-AIB1 cells was suppressed by CXCR4 silencing. Data are the mean ± SE of three independent experiments. **P < 0.01, *P < 0.05 versus cells transfected with A549-AIB1 by Student’s t test. **e** Upper left: the level of CXCR4 decreased by silence of AIB1, and then increased after the treatment of CXCR4 as detected by Western blotting. Upper right and down: Wound-healing assay showed that the attenuated migrative ability in H1993-shAIB1 cells was enhanced by the overexpression of CXCR4. **f** Transwell assay demonstrated that the attenuated invasive capacity of H1993-shAIB1 cells was enhanced by the overexpression of CXCR4. Data are the mean ± SE of three independent experiments. **P < 0.01, *P < 0.05 versus cells transfected with AIB1-shNC by Student’s t test

To identify potential downstream targets regulated by AIB1 that were involved in lung adenocarcinoma cell invasion and/or metastasis, the mRNA expression profiles of shAIB1-transfected H1993 cells were compared with those of the control H1993 cells using a Human Tumor Metastasis RT2 Profiler TM PCR Array containing 84 cell metastasis-related genes. The results showed that a total of five downregulated genes (3.5-fold) were identified in shAIB1-transfected H1993 cells (Table 2, Additional file 1: Table S1). Subsequently, these five downstream targets (CXCR4, TNFSF10, MMP11, MMP2, and VEGFA) were selected and analyzed by Western blotting (Fig. 4a). Consistent with the mRNA expression in the real-time PCR array, decreased protein expression of CXCR4 was shown by Western blotting in H1993 cells after AIB1 knockdown (Fig. 4b).

**Table 2:** Differential expression of 5 metastasis-related genes in H1993-shAIB1 cells relative to expression in H1993-vector cells

Gene symbol	Gene name	Location	Fold change
CXCR4	Chemokine (C-X-C motif) receptor 4	2q21	− 22.49
TNFSF10	Tumor necrosis factor (ligand) superfamily, member 10	3q26	− 8.37
MMP11	Matrix metallopeptidase 11	22q11.23	− 4.63
MMP2	Matrix metallopeptidase 2	16q13-q21	− 4.31
VEGFA	Vascular endothelial growth factor A	6p12	− 3.85

### CXCR4 mediates the invasiveness of AIB1-induced lung adenocarcinoma

To determine whether CXCR4 is a functional downstream target involved in AIB1-induced lung adenocarcinoma cell aggressiveness, two RNAis were used to silence CXCR4 expression in AIB1-overexpressing A549 cells (Fig. 4c). We found that after the siCXCR4 treatment of A549-AIB1 cells, the AIB1-induced migration and invasive capacities of the A549 cells were dramatically inhibited (*P *< 0.05, Fig. 4c, d). In addition, we upregulated the expression of CXCR4 in H1993-shAIB1 cells and found that the attenuated migration and invasion cell abilities caused by AIB1 depletion were rescued by CXCR4 overexpression (*P *< 0.05, Fig. 4e, f).

### Enforced expression of AIB1 enhances the metastasis potential of the lung adenocarcinoma cell line mediated by CXCR4 in vivo

**Fig. 5:**
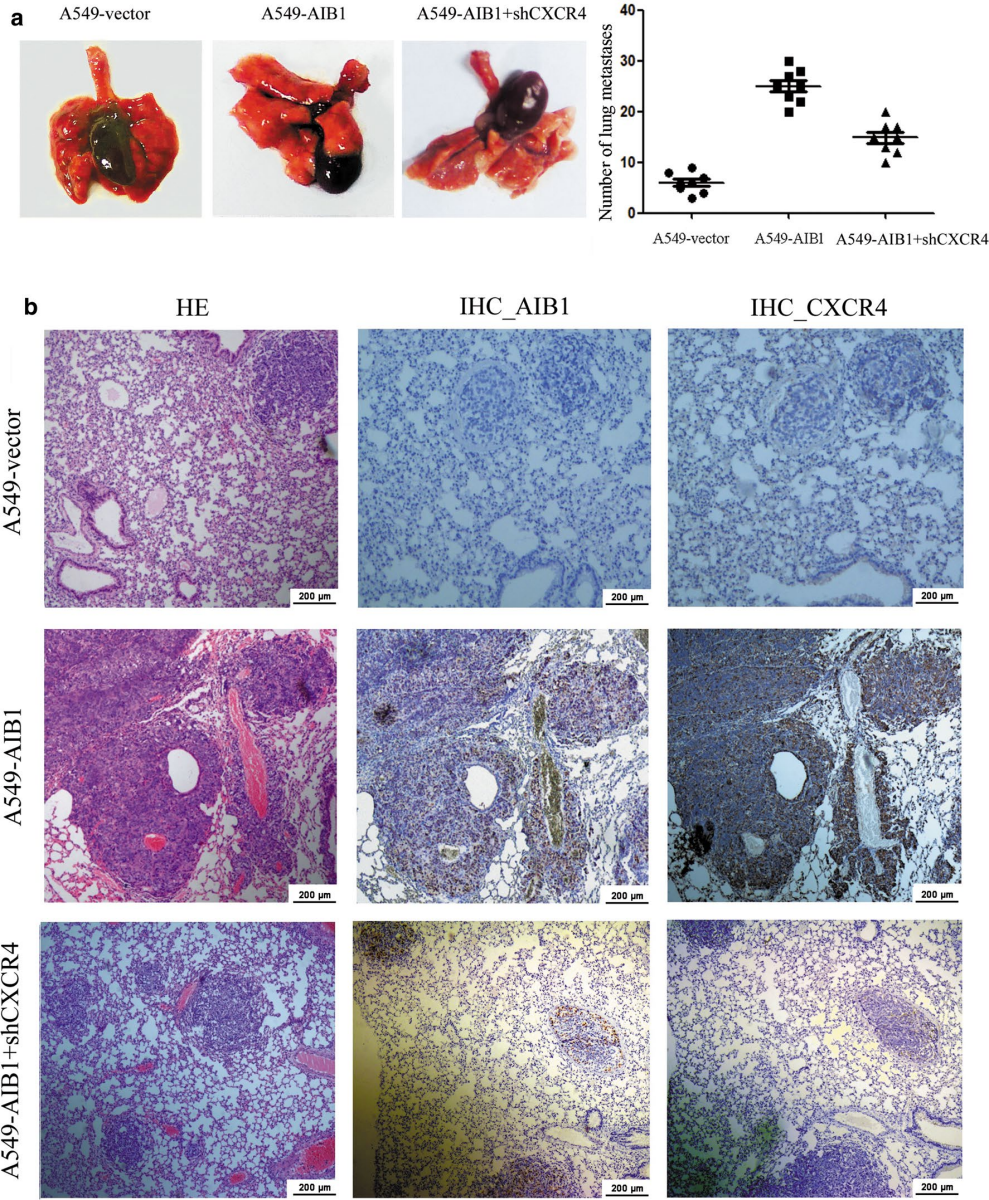
Overexpression of AIB1 enhances lung adenocarcinoma A549 cell metastasis mediated by CXCR4 in vivo. **a** Left: representative lungs showing metastatic nodules originating from A549-vector, A549-AIB1, and A549-AIB1 + CXCR4-shRNA cells injected with Balb/c nude mice. Right: number of metastatic nodules formed in the lungs of mice 6 weeks after tail vein injection of A549-vector, A549-AIB1, and A549-AIB1 + CXCR4-shRNA cells (eight mice per group; *P < 0.05; independent Student’s t test). **b** Representative hematoxylin and eosin (H&E) staining and IHC staining of AIB1 and CXCR4 in lung metastatic tumors originating from A549-vector, A549-AIB1, and A549-AIB1 + CXCR4-shRNA cells injected with Balb/c nude mice

To investigate whether AIB1 could affect the metastatic potential of lung adenocarcinoma cells in vivo and whether this effect could be mediated by CXCR4, we performed in vivo metastasis assays using a Balb/c nude mouse model. We did not detect any tumor nodules in the livers of all mice examined; however, metastatic tumor nodules were frequently found in the lungs of the mice (Fig. 5a). The expression levels of AIB1 and CXCR4, as detected by IHC, were simultaneously higher in the tumors of the A549-AIB1 group than in the tumors of the A549-vector group (Fig. 5b). The overexpression of AIB1 increased the number of lung metastases in mice injected with A549-AIB1 cells by approximately three-fold compared with the number in mice injected with A549 cells, whereas the depletion of CXCR4 dramatically decreased the number of AIB1-induced lung metastases (*P *< 0.05, Fig. 5b).

### Expression of CXCR4 in lung adenocarcinoma tissues and its correlation with AIB1 expression and patient survival

**Fig. 6:**
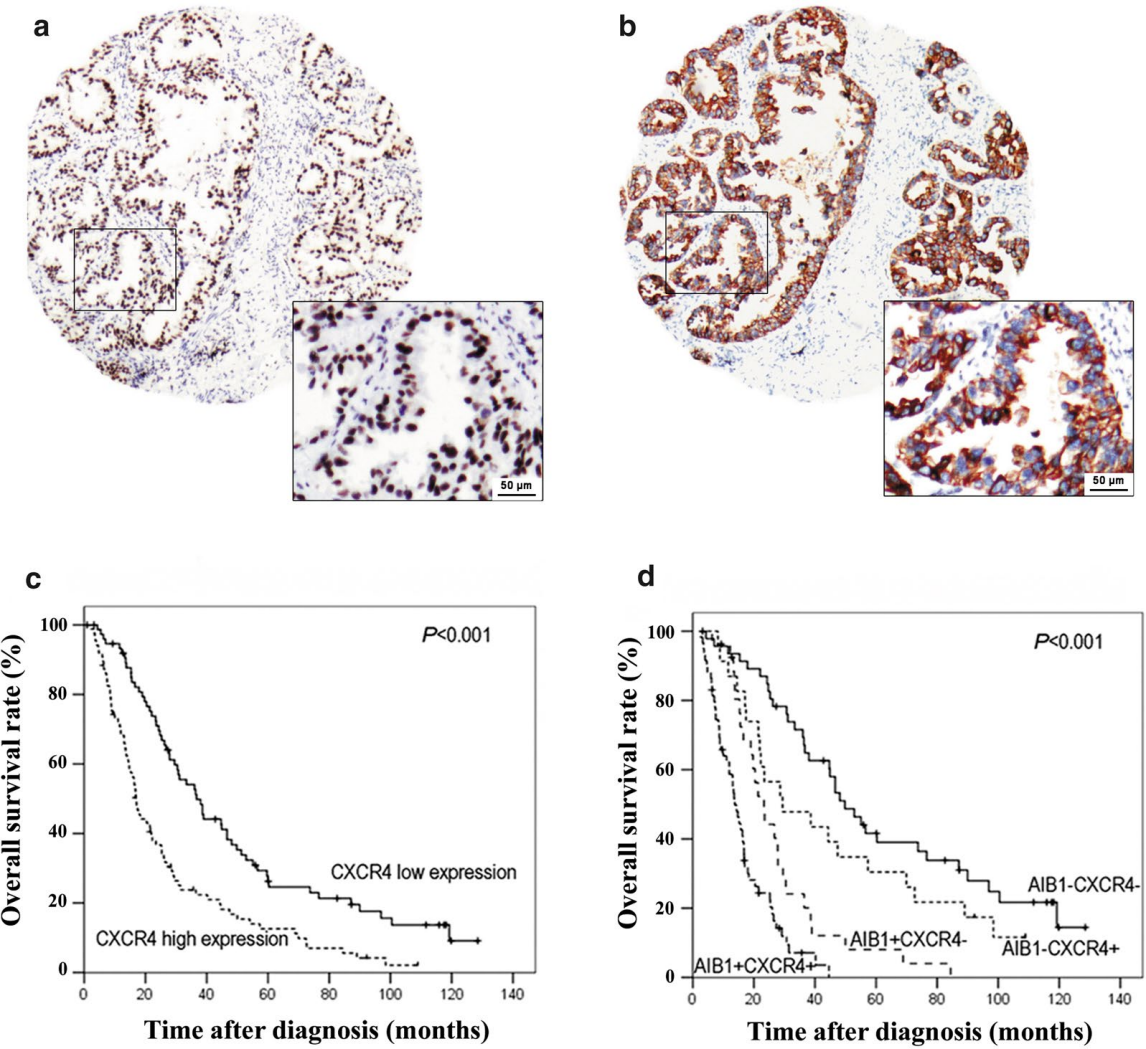
The associations of AIB1 and CXCR4 expression in patients with lung adenocarcinoma. **a** Overexpression of AIB1 and high-level expression of CXCR4 (**b**) were examined by IHC in a lung adenocarcinoma case. Overall survival curves according to the CXCR4 expression level (**c**) and both AIB1 and CXCR4 expression status (**d**) for lung adenocarcinoma patients

The median staining index of CXCR4 in lung adenocarcinoma was 3; thus, the categories of high and low expression were defined as groups with a staining index above or below 3. In 156 of the 183 samples, AIB1 and CXCR4 IHC were detected successfully and simultaneously. The rate of high CXCR4 expression was significantly greater in carcinomas overexpressing AIB1 (58/83 cases, 69.9%) than in those cases with a normal expression of AIB1 (23/73 cases, 31.5%, *P *< 0.001, Table 3, Fig. 6a, b). Furthermore, high expression of CXCR4 was significantly associated with poorer survival in lung adenocarcinoma patients (*P *< 0.001, Fig. 6c). In addition, lung adenocarcinoma patients with high expression of both AIB1 and CXCR4 displayed the poorest survival, whereas patients with low expression of AIB1 and CXCR4 had the best survival (*P *< 0.001, Fig. 6d).

**Table 3:** Associations of the expression status of AIB1 and CXCR4 in lung adenocarcinoma

CXCR4 expression	Cases	AIB1 expression [cases (%)]		*P*
		Normal	Over	
				< 0.001
Low	75	50 (66.7)	25 (33.3)	
High	81	23 (28.4)	58 (71.6)

## Discussion

In the present study, we report that the knockdown of AIB1 efficiently inhibits the migration and invasive abilities of lung adenocarcinoma in vitro, whereas the enforced overexpression of AIB1 substantially promotes lung adenocarcinoma migration and invasion in vitro and results in enhanced metastatic capacities in vivo. Importantly, we demonstrated that AIB1 enhances the migratory and metastasis abilities of lung adenocarcinoma cells by up-regulating the expression of chemokine receptor type 4 (CXCR4), an important downstream target. Furthermore, we showed that the simultaneous overexpression of AIB1 and CXCR4 predicts the poorest survival of LA patients.

Our previous study demonstrated the phenomenon of *AIB1* amplification in lung adenocarcinoma and showed that the overexpression of AIB1 was associated with pN status in M0 lung adenocarcinoma patients [13]. In the present study, we further found that the positive expression rate of AIB1 increased from the localized to regional to metastatic stages of lung adenocarcinoma tissues. Similar results were observed in other human cancers, such as breast [16], prostate [17, 18], esophageal [28], pancreatic [29], and colon/rectum cancer [15], in which overexpression of AIB1 was reported to be associated with lymph node metastasis and/or distant organ metastasis. In the present study, we demonstrated that AIB1 promotes lung adenocarcinoma migration in vitro and metastasis in vivo. These data support our emerging view that AIB1 is an important factor in promoting lung adenocarcinoma cell metastasis.

To the best of our knowledge, only two other studies have investigated the role of AIB1 in promoting cancer cell metastasis in vivo, one in breast cancer and the other in colorectal cancer, and only lung metastases were observed [15, 16]. Interestingly, in our mouse model, metastatic tumor nodules were also frequently found in lung tissues but not in liver tissues. It has been suggested that the expression of certain genes may lead to organ-specific metastasis in human cancers. For example, in colorectal cancer, the expression of transforming growth factor α (TGFα) often leads to liver-only metastasis [30], whereas in prostate cancer, the expression of platelet-derived growth factor receptor beta (PDGFR-β) often leads to bone-only metastasis [31]. These data suggest that AIB1 may promote lung metastasis in certain human cancers.

To date, the molecular mechanisms by which AIB1 promotes cancer cell migration/metastasis are not yet fully understood. In 2008, Qin et al. [16] first reported that AIB1 can promote breast cancer cell metastasis through matrix metalloproteinases (MMPs). Later, Long et al. [18] and Yan et al. [17] reported that AIB1 can promote prostate and breast cancer cell metastasis through focal adhesion kinase (FAK). More recently, Mo et al. [15] revealed that AIB1 promotes colorectal cancer by the Notch signaling pathway. However, little is known about the mechanism by which AIB1 promotes lung adenocarcinoma cell metastasis. To investigate the downstream molecular events involving AIB1 and lung adenocarcinoma metastasis, we compared the mRNA expression profiles of shAIB1-transfected H1993 cells and H1993-vector cells using a human tumor metastasis real-time PCR array. Of the 84 genes, 5 genes (CXCR4, TNFSF10, MMP11, MMP2, and VEGFA) showed differential expression of 3.5-fold or more at the mRNA level. Subsequently, downregulated *CXCR4* was validated in protein levels by Western blot. Furthermore, a positive correlation between the overexpression of AIB1 and CXCR4 was observed in our cohort of lung adenocarcinoma tissues. These results collectively suggest that AIB1 may promote lung adenocarcinoma cell metastasis by regulating CXCR4.

In recent years, CXCR4, which belongs to the family of chemokines, has been reported to be overexpressed and to play an important role in the cell proliferation, migration, and metastasis of several cancers, including non-small cell lung cancer [32, 33]. Regarding lung adenocarcinoma histologic subtypes, it has been reported that the cytomembranous expression of CXCR4 in lung adenocarcinoma is associated with metastasis and patient survival [34]. More recently, Bertolini et al. [35] demonstrated that the subset of CD133+/CXCR4+ lung adenocarcinoma cells are highly tumorigenic and metastatic in vivo. To determine whether CXCR4 is functionally involved in AIB1-induced lung adenocarcinoma cell aggressiveness, we silenced CXCR4 by using siRNA in A549-AIB1 cells. The results clearly showed that the silencing of CXCR4 substantially prevented AIB1-induced A549 cell migration and invasion. These data, taken together, indicate that AIB1 might promote lung adenocarcinoma cell metastasis by regulating CXCR4.

With respect to the potential molecular mechanisms of how AIB1 regulates the expression of CXCR4, Cheng et al. [36] reported that SDF-1α/CXCL12 induced cell migration via SRC-mediated CXCR4-EGFR cross-talk in gastric cancer cells. It has also been demonstrated that SRC regulates breast cancer cell proliferation and invasion through the autocrine/paracrine activity of SDF-1α/CXCL12. Stromal derived factor-1 (SDF-1α), also termed CXCL12, is the main ligand for CXCR4 [20]. However, CXCL12 has not been found to be obviously downregulated in our real-time PCR array. CXCR4 is one of the most important molecule in promoting metastasis [37]. Further studies are needed to elucidate the detailed mechanisms by which AIB1 regulates CXCR4 expression in lung adenocarcinoma.

In conclusion, our results provide evidence that (1) AIB1 promotes lung adenocarcinoma aggressiveness in vitro and in vivo by upregulating the expression of an important downstream target, CXCR4, and (2) AIB1 and CXCR4 may potentially serve as novel prognostic markers and/or therapeutic targets for this disease.

## Additional file

**Additional file 1: Table S1.** Expression of 84 metastasis-related genes in H1993-shAIB1 cells relative to that in H1993-vector cells.

## Abbreviations

AIB1: the amplified in breast cancer 1
LA: lung adenocarcinoma
CXCR4: chemokine receptor 4
TMA: tissue microarrays
IHC: immunohistochemistry
SCID: severe combined immune-deficient
ROC: receiver operating characteristic
TNFSF10: tumor necrosis factor superfamily, member 10
VEGFA: vascular endothelial growth factor A
TGFα: transforming growth α
PDGFR-β: platelet-derived growth factor receptor-β
MMP: matrix metalloproteinases
FAK: focal adhesion kinase
