# Supplementary material for: Overexpression of amplified in breast cancer 1 (AIB1) gene promotes lung adenocarcinoma aggressiveness in vitro and in vivo by upregulating C-X-C motif chemokine receptor 4
Source: Cancer Commun (Lond). 2018 Aug 13;38:53. doi: 10.1186/s40880-018-0320-1 (PMC6090807; doi:10.1186/s40880-018-0320-1)
Supplement: Supplementary file 1 — Additional file 1: Table S1. Expression of 84 metastasis-related genes in H1993-shAIB1 cells relative to that in H1993-vector cells. [file 40880_2018_320_MOESM1_ESM.docx]

**Table S1** Expression of 84 metastasis-related genes in H1993-shAIB1 cells relative to that in H1993-vector cells

| **Gene symbol** | **Gene name** | **Location** | **Fold change** | **Function** |
| --- | --- | --- | --- | --- |
| **Downregulated genes** | | | | |
| CD44 | CD44 molecule | 11p13 | -1.68 | Cell adhesion and stroma attachment |
| CD82 | CD82 molecule | 11p11.2 | -2.04 | Metastasis suppressor |
| CDH1 | Cadherin 1, type 1, E-cadherin (epithelial) | 16q22.1 | -1.33 | Inhibition of tumor metastasis |
| CDH11 | Cadherin 11, type 2, OB-cadherin (osteoblast) | 16q22.1 | -1.30 | Involved in the metastatic process |
| CDKN2A | Cyclin-dependent kinase inhibitor 2A | 9p21 | -1.47 | Negative regulation of cell cycle |
| CHD4 | Chromodomain helicase DNA binding protein 4 | 12p13 | -1.36 | Chromatin assembly and modification |
| COL4A2 | Collagen, type IV, alpha 2 | 13q34 | -1.09 | Component of extracellular matrix |
| CTBP1 | C-terminal binding protein 1 | 4p16 | -1.78 | Participates in cell adhesion |
| CTNNA1 | Catenin (cadherin-associated protein), alpha 1 | 5q31 | -1.57 | Protein hydrolysate |
| CTSK | Cathepsin K | 1q21 | -2.62 | Protein hydrolysate |
| CTSL | Cathepsin L1 | 9q21-q22 | -1.60 | Participates in cell adhesion |
| CXCL12 | Chemokine (C-X-C motif) ligand 12 | 10q11.1 | -1.42 | Participates in cell adhesion |
| CXCR2 | Chemokine (C-X-C motif) receptor 2 | 2q35 | -1.61 | Involved in tumor regulation and growth |
| CXCR4 | Chemokine (C-X-C motif) receptor 4 | 2q21 | -22.49 | Signal transduction, promotes invasion |
| EPHB2 | EPH receptor B2 | 1p36.1-p35 | -1.45 | Signal transduction, promotes invasion |
| ETV4 | Ets variant 4 | 17q21 | -2.36 | Transcription factor, promotes proliferation |
| FGFR4 | Fibroblast growth factor receptor 4 | 5q35.1 | -1.48 | Promotes invasion |
| FLT4 | Fms-related tyrosine kinase 4 | 5q34-q35 | -1.30 | Promotes tumor metastasis |
| FN1 | Fibronectin 1 | 2q34 | -1.72 | Participates in cell adhesion |
| FXYD5 | FXYD domain containing ion transport regulator 5 | 19q12-q13.1 | -1.18 | Negative regulation of cell adhesion |
| HGF | Hepatocyte growth factor | 7q21.1 | -1.30 | Participates in proteolysis, promotes proliferation |
| HPSE | Heparanase | 4q21.3 | -1.32 | Hydrolyses protein |
| HTATIP2 | HIV-1 Tat interactive protein 2 | 11q13 | -1.28 | Positively regulates transcription |
| IGF1 | Insulin-like growth factor 1 | 12q22-q23 | -1.54 | Promotes proliferation |
| IL18 | Interleukin 18 | 11q22.2-q22.3 | -2.84 | Promotes cell proliferation |
| IL1B | Interleukin 1, beta | 2q14 | -1.93 | Inhibits or promotes proliferation |
| ITGA7 | Integrin, alpha 7 | 12q13 | -3.37 | Participates in cell adhesion |
| ITGB3 | Integrin, beta 3 | 17q21.32 | -1.56 | Participates in cell adhesion |
| KISS1 | KiSS-1 metastasis suppressor | 1q32 | -1.23 | Suppresses metastasis |
| KISS1R | KISS1 receptor | 19p13.3 | -2.44 | Suppresses metastasis |
| KRAS | V-Ki-ras2 Kirsten rat sarcoma viral oncogene homolog | 12p12.1 | -1.54 | Cell signal transduction, proliferation |
| MCAM | Melanoma cell adhesion molecule | 11q23.3 | -2.70 | Participates in cell adhesion |
| MET | Met proto-oncogene | 7q31 | -1.29 | Proto-oncogene, promote cell proliferation |
| METAP2 | Methionyl aminopeptidase 2 | 12q22 | -2.20 | Protein hydrolysate and modification |
| MGAT5 | Mannosyl (alpha-1,6-)-glycoprotein beta-1,6-N-acetyl-glucosaminyltransferase | 2q21 | -1.01 | Promotes metastasis |
| MMP10 | Matrix metallopeptidase 10 | 11q22.3 | -1.30 | Protein hydrolysate, promote metastasis |
| MMP11 | Matrix metallopeptidase 11 | 22q11.23 | -4.63 | Decomposes protein hydrolysate and promotes metastasis |
| MMP13 | Matrix metallopeptidase 13 | 11q22.3 | -2.20 | Decomposes protein hydrolysate and promotes metastasis |
| MMP2 | Matrix metallopeptidase 2 | 16q13-q21 | -4.31 | decomposes protein hydrolysate and promotes metastasis |
| MMP3 | Matrix metallopeptidase 3 | 11q22.3 | -1.30 | Decomposes protein hydrolysate and promotes metastasis |
| MMP9 | Matrix metallopeptidase 9 | 20q11.2-q13.1 | -2.50 | decomposes protein hydrolysate and promotes metastasis |
| MTA1 | Metastasis associated 1 | 14q32.3 | -1.92 | Promotes metastasis |
| MYC | V-myc myelocytomatosis viral oncogene homolog | 8q24.12-24.13 | -1.04 | Promotes proliferation |
| MYCL | V-myc myelocytomatosis viral oncogene homolog 1, lung carcinoma derived | 1p34.2 | -2.62 | Transcription factor, promotes proliferation |
| NR4A3 | Nuclear receptor subfamily 4, group A, member 3 | 9q22 | -1.59 | Transcription factor, promotes proliferation |
| PLAUR | Plasminogen activator, urokinase receptor | 19q13 | -1.65 | Activator of plasminogen |
| PTEN | Phosphatase and tensin homolog | 10q23.3 | -2.08 | Inhibits proliferation and metastasis |
| RB1 | Retinoblastoma 1 | 13q14.2 | -1.69 | Negative regulation of cell reproduction |
| RORB | RAR-related orphan receptor B | 9q22 | -1.30 | Participates in regulate of transcription |
| SERPINE1 | Serpin peptidase inhibitor, clade E (nexin, plasminogen activator inhibitor type 1), member 1 | 7q22.1 | -1.18 | Inhibitor of fibrinolysis |
| SMAD2 | SMAD family member 2 | 18q21.1 | -1.21 | Cell signal transduction |
| SRC | V-src sarcoma (Schmidt-Ruppin A-2) viral oncogene homolog | 20q12-q13 | -2.20 | Promotes proliferation |
| SYK | Spleen tyrosine kinase | 9q22 | -1.12 | Promotes proliferation |
| TCF20 | Transcription factor 20 | 22q13.3 | -1.02 | Transcription factor |
| TGFB1 | Transforming growth factor, beta 1 | 17q25 | -2.51 | Inhibits or promotes proliferation, promotes metastasis |
| TIMP2 | TIMP metallopeptidase inhibitor 2 | 22q12.3 | -1.95 | Inhibits metastasis |
| TIMP3 | TIMP metallopeptidase inhibitor 3 | 3p25 | -1.94 | Induces apoptosis, inhibits metastasis |
| TIMP4 | TIMP metallopeptidase inhibitor 4 |  | -1.15 | Inhibits metastasis |
| TNFSF10 | Tumor necrosis factor (ligand) superfamily, member 10 | 3q26 | -8.37 | Induces apoptosis, inhibits proliferation |
| TP53 | Tumor protein p53 | 17p13.1 | -1.43 | Inducts apoptosis and cell differentiation, inhibits proliferation |
| TRPM1 | Transient receptor potential cation channel, subfamily M, member 1 | 15q13-q14 | -1.30 | Calcium channels |
| TSHR | Thyroid stimulating hormone receptor | 14q31 | -3.36 | Promotes proliferation |
| VEGFA | Vascular endothelial growth factor A | 6p12 | -3.85 | Promotes proliferation, metastasis; inhibits apoptosis |
| B2 M | Beta-2-microglobulin | 15q21-q22.2 | -2.64 | Immune response, MHC I receptor |
| GAPDH | Glyceraldehyde-3-phosphate dehydrogenase | 12p13 | -1.06 | Glycometabolism |
| RPLP0 | Ribosomal protein, large, P0 | 12q24.2 | -1.09 | Consists of a small 40S subunit and a large 60S subunit |
| **Upregulated genes** | |  |  |  |
| APC | Adenomatous polyposis coli | 5q21-q22 | 1.25 | Cell adhesion, inhibits proliferation |
| BRMS1 | Breast cancer metastasis suppressor 1 | 11q13-q13.2 | 1.62 | Cell adhesion and stroma attachment |
| CCL7 | Chemokine (C-C motif) ligand 7 | 16q13 | 1.63 | Promotes invasion and migration |
| CDH6 | Cadherin 6, type 2, K-cadherin (fetal kidney) | 5p15.1-p14 | 1.66 | Osteosis, cell adhesion |
| CST7 | Cystatin F (leukocystatin) | 20p11.21 | 1.01 | Inhibits cysteine proteinase |
| DENR | Density-regulated protein | 12q24.31 | 1.15 | Promotes proliferation |
| EWSR1 | Ewing sarcoma breakpoint region 1 | 22q12 | 1.54 | Transcription factor, promotes oncogenesis |
| FAT1 | FAT tumor suppressor homolog 1 | 4q35 | 1.77 | Participates in cell adhesion |
| GNRH1 | Gonadotropin-releasing hormone 1 | 8p21-p11.2 | 1.10 | Inhibits cell proliferation |
| HRAS | V-Ha-ras Harvey rat sarcoma viral oncogene homolog | 11p15.5 | 2.44 | Promotes proliferation |
| MDM2 | Mdm2 p53 binding protein homolog | 12q14.3-q15 | 1.16 | Negative regulation of cell proliferation |
| MMP7 | Matrix metallopeptidase 7 | 11q21-q22 | 2.50 | Decomposes protein hydrolysate and promotes metastasis |
| MTSS1 | Metastasis suppressor 1 | 8p22 | 1.78 | Inhibits metastasis and proliferation |
| NF2 | Neurofibromin 2 | 22q12.2 | 1.47 | Inhibits proliferation |
| NME1 | Non-metastatic cells 1 | 17q21.3 | 2.10 | Negatively regulates proliferation and participates in cell adhesion |
| NME4 | Non-metastatic cells 4 | 16p13.3 | 1.30 | Inhibits proliferation |
| PNN | Pinin, desmosome-associated protein | 14q21.1 | 1.06 | Inhibits proliferation |
| RPSA | Ribosomal protein SA | 3p22.2 | 1.00 | Participates in cell adhesion |
| SET | SET nuclear oncogene | 9q34 | 1.01 | Inhibits histone acetylate |
| SMAD4 | SMAD family member 24 | 18q21.1 | 1.13 | Cell signal transduction |
| SSTR2 | Somatostatin receptor 2 | 17q24 | 1.22 | Inhibits proliferation |
| ACTB | Actin, beta | 7p15-p12 | 1.79 | Forms cytoskeleton |
| HPRT1 | Hypoxanthine phosphoribosyltransferase 1 | Xq26.1 | 1.15 | Generates purine nucleotides through the purine salvage pathway |
